# Structural and Functional Integration of Tissue-Nonspecific Alkaline Phosphatase Within the Alkaline Phosphatase Superfamily: Evolutionary Insights and Functional Implications

**DOI:** 10.3390/metabo14120659

**Published:** 2024-11-25

**Authors:** Iliass Imam, Gilles Jean Philippe Rautureau, Sébastien Violot, Eva Drevet Mulard, David Magne, Lionel Ballut

**Affiliations:** 1 Molecular Microbiology and Structural Biochemistry, UMR 5086, CNRS, University Lyon, F-69367 Lyon, France; iliass.imam@ibcp.fr (I.I.); sebastien.violot@univ-lyon1.fr (S.V.); 2 Institute of Chemistry and Biochemistry (ICBMS), UMR 5246, CNRS, University Lyon, F-69622 Villeurbanne, France; gilles.rautureau@univ-lyon1.fr (G.J.P.R.); eva.drevet-mulard@univ-lyon1.fr (E.D.M.)

**Keywords:** tissue-nonspecific alkaline phosphatase (TNAP), alkaline phosphatase (AP) superfamily, ectonucleotide pyrophosphatases/phosphodiesterases (ENPP), arylsulfatases (ARS)

## Abstract

Phosphatases are enzymes that catalyze the hydrolysis of phosphate esters. They play critical roles in diverse biological processes such as extracellular nucleotide homeostasis, transport of molecules across membranes, intracellular signaling pathways, or vertebrate mineralization. Among them, tissue-nonspecific alkaline phosphatase (TNAP) is today increasingly studied, due to its ubiquitous expression and its ability to dephosphorylate a very broad range of substrates and participate in several different biological functions. For instance, TNAP hydrolyzes inorganic pyrophosphate (PP_i_) to allow skeletal and dental mineralization. Additionally, TNAP hydrolyzes pyridoxal phosphate to allow cellular pyridoxal uptake, and stimulate vitamin B6-dependent reactions. Furthermore, TNAP has been identified as a key enzyme in non-shivering adaptive thermogenesis, by dephosphorylating phosphocreatine in the mitochondrial creatine futile cycle. This latter recent discovery and others suggest that the list of substrates and functions of TNAP may be much longer than previously thought. In the present review, we sought to examine TNAP within the alkaline phosphatase (AP) superfamily, comparing its sequence, structure, and evolutionary trajectory. The AP superfamily, characterized by a conserved central folding motif of a mixed beta-sheet flanked by alpha-helices, includes six subfamilies: AP, arylsulfatases (ARS), ectonucleotide pyrophosphatases/phosphodiesterases (ENPP), phosphoglycerate mutases (PGM), phosphonoacetate hydrolases, and phosphopentomutases. Interestingly, TNAP and several ENPP family members appear to participate in the same metabolic pathways and functions. For instance, extra-skeletal mineralization in vertebrates is inhibited by ENPP1-mediated ATP hydrolysis into the mineralization inhibitor PP_i_, which is hydrolyzed by TNAP expressed in the skeleton. Better understanding how TNAP and other AP family members differ structurally will be very useful to clarify their complementary functions. Structurally, TNAP shares the conserved catalytic core with other AP superfamily members but has unique features affecting substrate specificity and activity. The review also aims to highlight the importance of oligomerization in enzyme stability and function, and the role of conserved metal ion coordination, particularly magnesium, in APs. By exploring the structural and functional diversity within the AP superfamily, and discussing to which extent its members exert redundant, complementary, or specific functions, this review illuminates the evolutionary pressures shaping these enzymes and their broad physiological roles, offering insights into TNAP’s multifunctionality and its implications for health and disease.

## 1. Introduction

Tissue-nonspecific alkaline phosphatase (TNAP) is a ubiquitously expressed ectoenzyme that catalyzes the hydrolysis of various phosphorylated substrates. It is mainly known for its necessary role in stimulating bone mineralization. To achieve this function, TNAP is expressed in growth plate chondrocytes, osteoblasts and odontoblasts, where it hydrolyzes extracellular inorganic pyrophosphate (PP_i_), a constitutive inhibitor of soft tissue calcification, to allow the deposition of calcium phosphate apatite crystals within collagen fibrils [1]. Mutations in the ALPL gene encoding TNAP that cause a decrease in TNAP production, addressing and/or activity, lead to the genetic disease hypophosphatasia (HPP), mainly characterized by an impairment of bone mineralization, which can result in the most severe forms in the perinatal death of babies with almost no mineral in their skeleton [2]. Besides mineralization, TNAP is also required for the dephosphorylation of pyridoxal phosphate (PLP) into pyridoxal (PL), allowing PL to enter cells to support vitamin B6-dependent reactions. Consequently, TNAP-deficient mice and individuals with HPP also suffer from epileptic seizures, due to a lack of vitamin B6-dependent synthesis of the inhibitory neurotransmitter gamma-aminobutyric acid (GABA) [3]. Moreover, TNAP is able to dephosphorylate adenosine triphosphate (ATP) and lipopolysaccharide (LPS), acting as an anti-inflammatory and immunomodulatory protein [4,5,6]. In addition to these acknowledged substrates and functions of TNAP, several other phosphorylated compounds were recently proposed to be dephosphorylated by TNAP, further broadening TNAP’s multisystemic functions [7]. For instance, recent studies suggested the implication of TNAP in adaptive thermogenesis, specifically in creatine futile cycling, where phosphocreatine, synthesized by type B creatine kinase, would be hydrolyzed by TNAP, producing heat during the process [8]. It is likely that TNAP exerts other crucial functions, which so far remained unknown due to the fact that severe TNAP deficiencies are lethal both in humans and mice. One way to discover new TNAP substrates and functions may be to explore the three-dimensional structure of its active site and how it may catalyze the dephosphorylation of different substrates. The 3D structure of TNAP has indeed been very recently published [9]. Such experimental approaches have provided very interesting information concerning the possible substrates of a subfamily within the alkaline phosphatase (AP) superfamily, the ectonucleotide pyrophosphatase phosphodiesterase (ENPP) [10].

TNAP is part of the AP superfamily, in which the members share a conserved catalytic site, with specificities that affect substrate affinity and enzymatic promiscuity [9,10]. The AP superfamily can be divided into six different subfamilies: AP (3.1.3.1), ENPP (3.1.4.1), phosphoglycerate mutases (PGM) (5.4.2.1), phosphonoacetate hydrolases (3.11.1.2), phosphopentomutases (5.4.2.7) and arylsulfatases (ARS) (3.1.6.8). These enzymes function at high pH and can hydrolyze many substrates. In the case of the arylsulfatases, their preferential substrates are sulfated but they can also hydrolyze phosphate esters with lesser activity, as phosphate and sulfate share similar chemical properties. As arylsulfatases share the same catalytic site as the other members of the AP superfamily, they were classified as part of this group of enzymes.

This review aims to explore the similarities and differences, in terms of sequence and structure, between TNAP and other AP superfamily members, to better understand the interrelationships between these enzymes, mainly in vertebrates. We will focus on AP, ENPP and ARS, and will not present data on the three other subfamilies. The different members of AP, ENPP and ARS subfamilies, in humans and mice, are presented in Table 1.

## 2. The Superfamily of Alkaline Phosphatases, a Conserved Central Folding

Members of the AP superfamily share a conserved central folding organized around a central mixed beta-sheet with seven (eight) beta strands surrounded by six (seven) alpha helices [11,12,13,14,15,16]. This AP core corresponds to the Interpro signature IPR017850. The AP superfamily currently includes six families: AP (3.1.3.1), ARS (3.1.6.8), ENPP (3.1.4.1), phosphoglycerate mutases (5.4.2.1), phosphonoacetates (3.11.1.2), and phosphopentomutase (5.4.2.7). Only the first three families are found in humans, with the other three being primarily prokaryotic [17].

**Figure 1:**
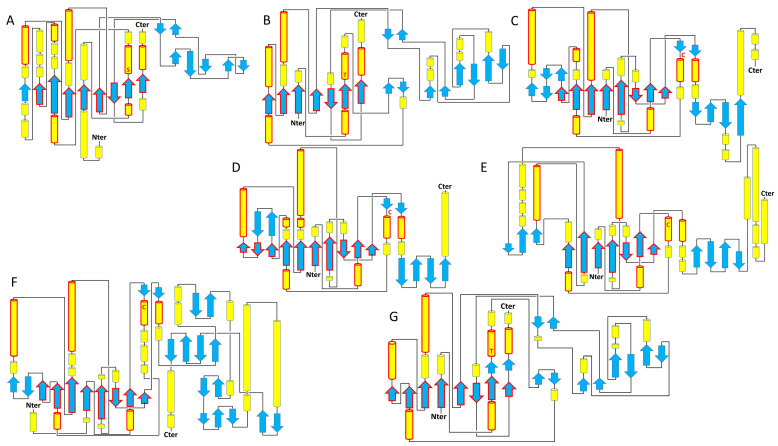
Topology of representative members of the AP superfamily. (**A**) Human TNAP, (**B**) human ENPP5, (**C**) human ARSA, (**D**) human ARSB, (**E**) human ARSK, (**F**) human SULF1, (**G**) human ENPP1. The central core is conserved in members of the AP, ENPP, and ARS families, with a minimal conservation of 7 to 8 beta strands and 6 to 7 alpha helices highlighted in solid red lines. The additional domains of ENPP1, somatomedin at the N-terminus (1–192), and non-specific DNA/RNA endonuclease at the C-terminus (637–925) are not shown for clarity. The secondary structures, alpha helices, and beta strands are shown in yellow and blue, respectively. The catalytic residues are indicated by the letters S (serine), T (threonine), and C (cysteine).

For human TNAP (PDB codes: 7YIV, 7YIW, 7YIX), the conserved mixed central sheet is organized in the arrangement (4)↑3↑5↑1↑6↑7↓2↑8↑ [9]. A CATH analysis conducted on all AP members in humans confirms this structural classification with the AP core belonging to the alpha/beta class, with a three-layer (aba) Sandwich architecture, and an alkaline phosphatase-type topology [18]. The topologies of seven representative members of ARS, ENPP, and AP obtained from PDBsum consistently show the presence of this central core, albeit with very different organizations outside of it (Figure 1) [19]. For example, ARSK (Uniprot: Q6UWY0) or ENPP5 (PDB code: 5VM) present a minimal central beta-sheet with seven beta-strands, while ARSA (PDB code: 1AUK), ARSB (PDB code: 1FSU), or SULF1 (Uniprot: Q8IWU6) have this central beta-sheet stabilized by three additional strands, TNAP and ENPP1 (PDB code: 6WET), presenting nine and eight beta strands, respectively (Figure 1). Yu et al. provided a detailed description of the TNAP structure. In addition to the central beta-sheet, the authors identify two clusters of alpha helices: one called CA, which accommodates the Ca^2+^ ion in the structure, and another named MZ, which holds the Mg^2+^/Zn^2+^ ions at the catalytic site. They note that the structure is highly conserved across alkaline phosphatases, with a root-mean-square deviation (RMSD) of 1.625 Å between human TNAP and human PLAP. A distinctive feature of human phosphatases, compared to *E. coli* alkaline phosphatase, is the presence of an N-terminal extension composed of helices and loops. This crown-like domain stabilizes the interface [9,13].

**Figure 2:**
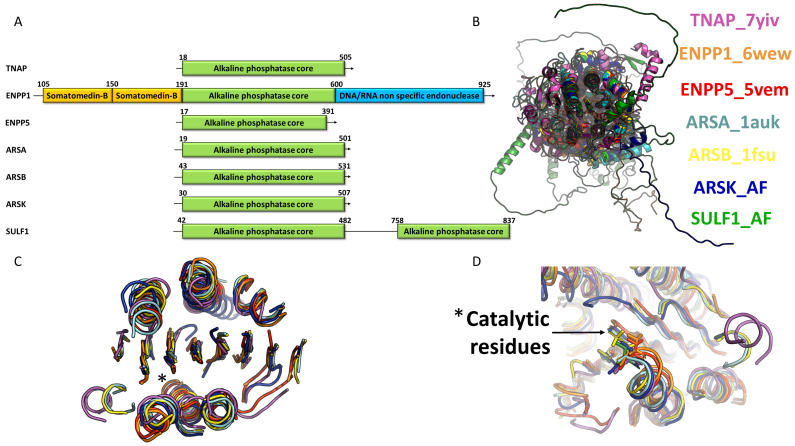
Conservation of the core in the AP superfamily. (**A**) Examples of domain organization in various members of the superfamily. (**B**) Superposition of 7 representative members of the superfamily, including AP, ENPP, and ARS. The members are TNAP (ALPL) in pink (PDB code: 7YIV), ENPP1 in orange (PDB code: 6WEW), ENPP5 in red (PDB code: 5VEM), ARSA in light blue (PDB code: 1AUK), ARSB in yellow (PDB code: 1FSU), ARSK in dark blue (AlphaFold model), and SULF1 in light green (AlphaFold model). The superposition focuses solely on the central core, highlighting the extreme organizational variability and the lack of overall folding conservation outside the catalytic core. (**C**) Extraction of the minimal secondary structures from the previous superposition that constitutes the central core. This reveals the high conservation of the central core’s folding, with its 7 to 8 beta strands and 6 to 7 alpha helices. The star marks the position of the nucleophilic catalytic residue: serine for TNAP, threonine for ENPP1 and ENPP5, modified cysteine for ARSA (formylglycine) and ARSB (sulfate ester), and cysteine for the AlphaFold models of ARSK and SULF1. (**D**) Conserved positioning of the catalytic residue at the end of the helix.

Generally, around this minimal organization of seven (eight) beta strands and six (seven) alpha helices, there are more or less supplementary secondary structures, with the size of the superfamily members varying from 440 residues for ENPP6 (Uniprot: Q6UWR7) to 925 residues for ENPP1 (Uniprot: P22413) [16]. However, despite important size differences, an InterPro or CATH analysis only reveals a few additional conserved domains for ARS and ENPP members, with AP possessing only the alkaline phosphatase core domain. An excellent review by Borza et al. indeed recalls that ENPP1, 2 and 3 have two somatomedin B domains at the N-terminus and a non-specific DNA/RNA endonuclease domain at the C-terminus (Figure 1) [10,20,21,22]. It is worth noting that this DNA/RNA endonuclease domain does not appear to be functional and could contribute to stabilizing the AP domain. Meanwhile, it is observed that ENPP4-7 all have a minimal organization with only the central core alkaline phosphatase domain [23,24,25] (Figure 2A).

For the arylsulfatase family, most of the 17 members [ARSA, B, C (sterylsulfatase: STS), D, F, H, I, J, L, and GALNS (N-acetylgalactosamine 6-sulfatase)] have a very short conserved domain at the C-terminus with a two-layer sandwich architecture [11,12,14,15,16,26]. SULF1 (Uniprot: Q8IWU6) and SULF2 (Uniprot: Q8IWU5) do not have this domain but present a very long extension at the C-terminus of the AP domain, which could be described as a hydrophilic HD domain [27,28]. However, this HD domain does not exhibit a characteristic InterPro, CATH, or Pfam signature due to sequence variability [29]. Similarly, an additional region, referred to as subdomain 2, is observed in iduronate 2-sulfatase (IDS), which consists of four antiparallel strands. This region is longer than the beta meander motif found in counterparts such as GALNS [16]. A superposition of experimentally obtained structures by X-ray crystallography (TNAP, ENPP1, etc.) or predicted by AlphaFold (ARSK and SULF1) confirms that, outside the AP core domain whose secondary structures perfectly align (Figure 2C), members of the superfamily fold in very different ways (Figure 2B). Thus, this central fold seems to have undergone strong selection pressure, suggesting that this organization likely has relevance for the hydrolysis of sulfo- and phosphoester bonds. This conserved organization of the AP core is further evidenced by the fact that the catalytic nucleophile residues [serine for AP and ENPP6 [9,10,13,30], threonine for ENPP [10,31], and cysteine (formylglycine) for ARS [32]] are systematically present at the end of the alpha helix in the C-terminal position of beta-strand 2, alpha helix 2 for TNAP (Figure 2D). It is important to note that in available structures, the catalytic residue is not only carried by the same conserved helix, but it is also consistently positioned in the same location with the nucleophilic group oriented similarly (Figure 2D). Overall, the three alkaline phosphatase families presented here all share a structurally conserved catalytic domain and general folding, with a nucleophilic catalytic residue specific to each family.

## 3. The Oligomeric State of Members of the Alkaline Phosphatase Superfamily

**Figure 3:**
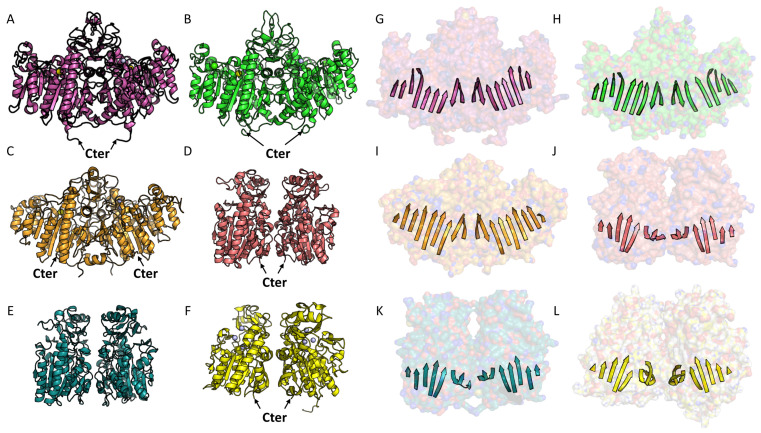
Comparison of dimeric organization in AP and ENPP. (**A**–**F**) Examples of characteristic dimers of AP and ENPP. (**G**–**L**) Organization of the central β-sheet characteristic of the AP family. The Van der Waals surface of the dimer is shown in transparency. The dimeric organization is consistent across the AP family, including human enzymes like TNAP (PDB code: 7YIV) (panels **A**,**G**) and PLAP (PDB code: 1ZED) (panels **B**,**H**), as well as the *E. coli* AP enzyme (panels **C**,**E**). This organization features conserved central β-sheets, typical of the AP fold, facing each other without complementary interactions (panels **G**,**H**,**E**). A similar AP-like interface is observed in some ENPP family members, particularly in murine ENPP6 (panel **D**) (PDB code: 5EGH). An AlphaFold model of the human ENPP6 dimer suggests a similar organization (panel **F**) (PDB code: 5VEN). The same AP-like interface is observed in murine ENPP5, with the conserved central β-sheets facing each other with a slight offset and no complementary interactions (panels **J**,**K**,**L**).

The oligomeric state of members of the AP superfamily is not always known, as the characterization of this state is modulated by various cellular or tissue factors [9,10,26,33,34,35]. Regarding APs, a minimal organization is found in the form of a homodimer, at least for TNAP and placental alkaline phosphatase (PLAP), and a conserved dimeric interface is observed for these two enzymes, as seen in the crystal structures (PDB code: 7yiv; PDB code: 1ZEF) (Figure 3A,B,G,H) [9,13,30,33,36]. Le Du and Millan report that the interaction surface in alkaline phosphatase ranges from 4134 to 4244 Å2 per monomer, representing 25% of the total surface and involving 90 residues [33]. Additionally, Yu et al. show that the TNAP interface is mainly composed of hydrophobic interactions and hydrogen bonds, with a single salt bridge between arginine 71 and aspartate 458. Confirming the importance of this interface, missense mutations among residues involved in these interactions, notably R71, have been observed in patients with hypophosphatasia (HPP), resulting in dominant negative effect and symptoms ranging from moderate to severe [37,38,39]. A dimer and a similar interface are also found for the alkaline phosphatase of *E. coli* (Figure 3C,I) [9,40,41]. The conservation of this interface suggests an important role in enzyme stabilization and its catalytic process [33]. As for TNAP, it is proposed that it can form higher-order oligomers in addition to its dimeric state [42]. A homo-octamer with two distinct interfaces, termed L (large) and S (small), has been observed in the crystal structure of TNAP [9]. The exact function of this higher-order oligomeric state is not completely understood but natural mutants associated with the genetic metabolic disorder hypophosphatasia are localized at these two interfaces [9,43,44]. This suggests that beyond dimerization, octamerization or higher-order oligomerization is crucial for maintaining the enzyme’s structure and activity. Interestingly, Le Du and Millán investigated the dimeric interface of the different members of the APs. They showed that TNAP/GCAP (germ cell alkaline phosphatase) and PLAP/IAP (intestinal alkaline phosphatase) heterodimers were not only possible but also physiologically relevant as they are observed in human postnatal intestine- and ovarian cancer-derived cell lines, respectively [33].

Regarding the ENPP enzymes, we observe quite a variable oligomeric organization, since a dimer is clearly identified for ENPP1 and ENPP3 but not for other human members of the family [21,22]. However, it is worth noting that despite the conserved central folding, ENPP1 and ENPP3 present a dimeric interface completely different from that of AP [10]. ENPP1 and ENPP3 are single-pass type II membrane proteins that can be secreted following proteolytic cleavage, in contrast to ENPP2, which is a secreted monomeric enzyme [45].

Regarding the other human ENPP4, 5 and 7, their biochemical and biophysical characterization has not revealed homologous interaction phenomena [46,47,48]. No structure is available for human ENPP6; however, two structures exist for mouse ENPP6 (PDB codes: 5EGE, 5EGH). Mouse ENPP6 was purified as a monomer, dimer, and higher-order oligomer, and a dimer was found in the asymmetric unit after crystallization, showing an interface similar to that observed for TNAP or PLAP, with the two central beta sheets slightly offset (Figure 3D,J) [49]. A model of the human ENPP6 dimer obtained using AlphaFold reveals the same type of dimerization (Figure 3E,K) [50]. Regarding ENPP5, structures for both the human and mouse enzymes exist (PDB codes: 5VEM and 5VEN, respectively) [47]. The authors report that both enzymes were purified in monomeric form. However, it is interesting to note that while the human ENPP5 structure does not show an AP-like dimeric interface, the mouse ENPP5 forms a homodimer in the asymmetric unit, identical to the mouse ENPP6 (Figure 3F,L). Finally, regarding ENPP4 and ENPP7, structures exist only for the human enzymes (PDB codes: 4LR2 and 5TCD, respectively) [46,48]. However, no AP-like dimeric interface is observed in these structures. Altogether, it appears that the same dimerization interface has been conserved throughout evolution for AP members as well as for murine ENPP5 and ENPP6. It remains to be proven whether human ENPP5 and ENPP6 can exhibit the same. Therefore, one may wonder if, more generally, the ENPPs could have their activity modulated by oligomerization processes.

**Figure 4:**
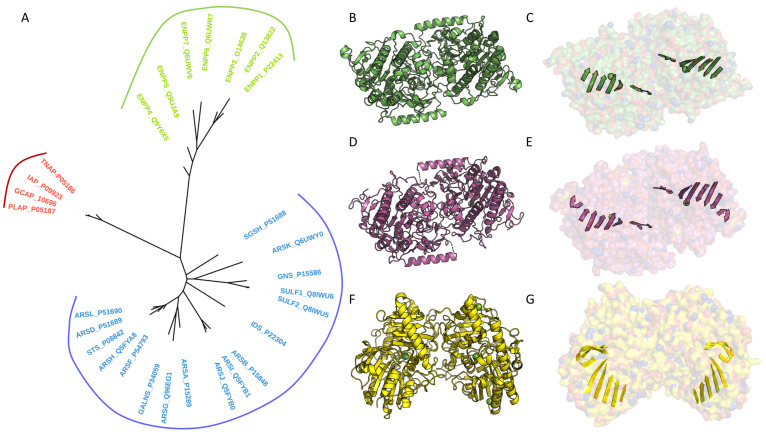
Evolution of members of the AP superfamily: (**A**) Phylogenetic tree of the AP superfamily members. Multiple sequence alignment of all human members of the AP superfamily was performed with the MAFFT program version 7 [54]. The sequences used were retrieved from the UniProtKB database, with the corresponding UniProt codes listed next to the enzyme names. Conserved blocks were selected by using BMGE1.12 [55] and the BLOSUM30 [56] matrix. Two hundred sites were kept for further analysis after character trimming was performed by BMGE [55]. Phylogenetic analyses were performed, with the LG model and a gamma correction, using a bootstrapped maximum-likelihood approach with PhyML 3.0 [57]. The phylogenetic tree was generated and visualized with iTOL software Version 6.9.1 [58]. (**B**,**D**,**F**) Examples of characteristic dimers observed in ARS. (**C**,**E**,**G**) Central β-sheet illustrating the diversity of organization in ARS. The Van der Waals surface of the dimer is shown in transparency. Two types of organization are presented here: one corresponding to GALNS (PDB code: 4FDJ) and ARSA (PDB code: 1AUK) (**B**–**E**) and the other to N-sulphoglucosamine sulphohydrolase (SGSH; PDB code: 4MHX) (**F**,**G**).

It is interesting to note that APs, as well as ENPP5 and ENPP6, are all membrane proteins. All APs undergo a maturation process during which their C-terminal end, which functions as a propeptide, is removed after proteolytic cleavage [10,51,52]. This cleavage is followed by a glypiation process that allows their anchoring to the membrane [53]. A similar process occurs for ENPP6, once again illustrating the functional and structural proximity of these two enzymes [10]. As for ENPP5, it has a C-terminal transmembrane helix that also allows its anchoring to the membrane [47]. Due to their structural proximity, it is observed that the orientation of the GPI anchors for APs and ENPP6, or the transmembrane helix for ENPP5, is precisely the same relative to the C-terminal orientation. This allows for the possibility of dimer formation at the membrane for all three (Figure 3A,D,F) [10]. In terms of evolution, the three families appear to form monophyletic groups. The shared characteristics observed between APs and certain ENPP members, such as the dimeric form, GPI anchor, and conserved catalytic core, suggest a possible common ancestor (Figure 4A).

Arylsulfatases are more heterogeneous in terms of size and sequence conservation than ENPPs and APs (Figure 2A). Their oligomeric states also vary significantly. ARSA forms an octamer at pH 5.0–5.4 and a dimer at pH 6.5, but with an interface different from those observed in APs or ENPP5 and ENPP6 (Figure 4B,C) [12,26,59]. In contrast, ARSB, ARSG, and IDS are present as monomers [11,16,60]. GALNS is also observed as a homodimer, which is identical to the dimer observed for ARSA (Figure 4B,C,D,E) [15]. SGSH also forms a dimer in the asymmetric unit, but with a unique organization and an interface distinct from those of ARSA or GALNS (Figure 4F,G) [61].

The oligomeric form of human ARSC is less well defined. Hernandez-Guzman et al. suggest that ARSC could exist as a monomer or dimer based on native PAGE analysis [14,62]. Later, Ghosh proposed the formation of a trimer associated with the membrane, with each monomer associated via pairs of antiparallel hydrophobic helices, as observed in the crystal structure (PDB code: 8EG3) [35]. Once again, multimerization appears to govern the stability and activity of arylsulfatases. Unfortunately, many of these enzymes have not yet been subjected to detailed biochemical or biophysical characterization, and numerous structures remain uncharacterized [26]. Overall, members of the alkaline phosphatase superfamily exist under a variety of oligomeric states and interfaces. However, within the AP family, including TNAP, members are mainly active in the form of hetero- or homodimers, and share a common interface that is essential for their biological function and structural integrity.

## 4. Catalytic Center Organization

**Figure 5:**
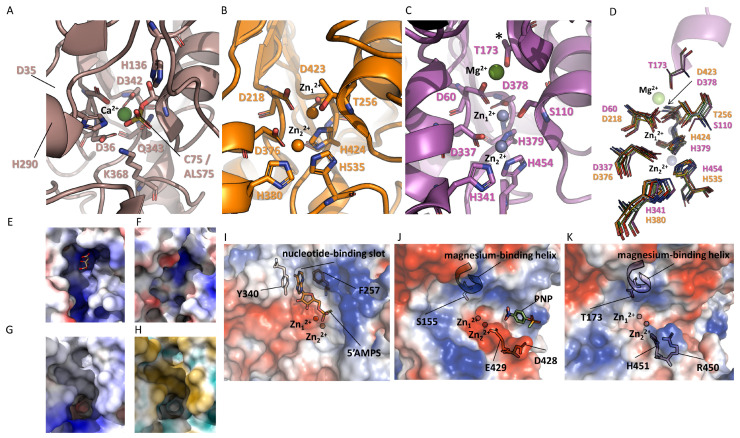
Comparison of the catalytic centers of AP, ENPP, and ARS, representative of each family. (**A**) Catalytic center of ARSC (PDB code: 8EG3). Conserved residues of the family are indicated. Calcium is shown as a green sphere. (**B**) Catalytic center of ENPP1 (PDB code: 6WEU). Conserved residues of the family are indicated. Zinc 1 and 2 are shown as orange spheres. (**C**) Catalytic center of TNAP (PDB code: 7YIV). Conserved residues of the family are indicated. Zinc 1 and 2 are shown as pink spheres, and magnesium is shown as a green sphere. (**D**) Superposition of representative members from the AP and ENPP families. Conserved residues between the two families are shown, indicating that the catalytic residue and the residues interacting with zinc are almost perfectly superimposable. The residues of TNAP and ENPP1 are numbered. The structures used in the comparison are TNAP in purple (PDB code: 7YIV), PLAP in light green (PDB code: 1ZED), ENPP1 in orange (PDB code: 6WEU), ENPP2 in yellow (PDB code: 5M7M), ENPP3 in light blue (PDB code: 6C02), ENPP4 in cyan (PDB code: 4LQY), ENPP5 in red (PDB code: 5VEM), and ENPP7 in dark green (PDB code: 5UDY). (**E**–**H**) Characteristic crevice of ARS with the catalytic pocket at its bottom. (**E**,**F**) Electrostatic surfaces of GALNS (PDB code: 4FDJ) and IDS (PDB code: 5FQL) reveal a highly positively charged crevice. A citrate molecule is visible at the bottom of the GALNS structure. (**G**,**H**) Electrostatic and hydrophobic surfaces of the binding site of ARSC (PDB code: 8EG3), showing one side that is positively charged (blue) (**G**) and the other side that is hydrophobic (yellow) (**H**). (**I**–**K**) Electrostatic surfaces of ENPP1, PLAP, and TNAP, with all three enzymes similarly oriented. Zn^2+^ ions are indicated by red and gray spheres. In ENPP1, adenosine-5′-thio-monophosphate is visible in the active site, with the base positioned in the nucleotide-binding slot stabilized by residues Y340 and F257 (**I**). In PLAP and TNAP, the magnesium-binding helix occupies the nucleotide-binding slot present in ENPP1, necessitating a different substrate accommodation. This is observed in PLAP (**J**) with the presence of PNP. The gatekeeper residues, D428 and E429 for PLAP and R450 and H451 for TNAP, are shown as sticks (**J**,**K**).

If the central folding exhibits a highly conserved secondary structure, it becomes evident that the catalytic sites have undergone divergent evolution, leading to the emergence of two prominent activities within this superfamily: phosphatase and sulfatase activities (Figure 5). In ENPPs, except for ENPP6, which features a serine, the catalytic residue is typically threonine, whereas in APs, it is serine [9,10,36]. Specifically, in sulfatases, the catalytic residues include a cysteine which undergoes post-translational modification to form formylglycine (FG) [32]. This FG is subsequently activated by a water molecule to yield hydroxyformylglycine (HFG).

When comparing the three families, there is a notable disparity in the presence of metal ions. Sulfatases exclusively harbor a single divalent cation, identified as Ca^2+^, which is hexacoordinated by three aspartates, one asparagine, and an oxygen atom derived from a sulfate group, forming an ester linkage with hydroxyformylglycine, as observed in the structure of ARSA (PDB code: 1N2K), ARSB (PDB code: 1FSU) or ARSC (STS) (PDB code: 8EG3) (Figure 5A) [11,35,59]. The residues involved in this coordination are fully conserved in human sulfatases [63,64].

ENPPs have two metallic ions at their active site. These consist of two zinc ions coordinated, respectively, by two aspartates, one histidine, and the nucleophilic catalytic residue for the first (Zn1), and by two histidines and one aspartate for the second (Zn2). Again, these residues are completely conserved in human ENPPs (Figure 5B) [10]. It is noted that the calcium in sulfatases and zinc number 1 in ENPPs occupy the same position in the catalytic site. Two residues involved in coordination are perfectly conserved, such as residues D35 and D342 in ARSC, which correspond, respectively, to residues D218 and D423 in ENPP1 (PDB code: 6WEU). In sulfatases, a glutamine (Q343 in ARSC) is replaced by a histidine in ENPPs (H424 in ENPP1) (Figure 5A,B).

A single insertion stands out as the distinguishing feature between the catalytic sites of ENPPs and AP. This feature is a short alpha helix comprising six amino acids and forming 1.5 turns (helix 153–158 for PLAP and helix 171–176 for TNAP), positioned near the catalytic residue (Figure 5C). This helix harbors a threonine in PLAP and a serine in the other three APs, facilitating the coordination of a magnesium ion [9,13]. We will refer to this helix as the magnesium-binding helix. This coordination is complemented by two conserved aspartates in human APs and three water molecules, as evidenced in the structure of PLAP (PDB code: 1ZED) [30].

Magnesium is essential in APs, activating serine and participating in its deprotonation [65]. Furthermore, the presence of this Mg^2+^ ion elucidates the substitution of a serine for ENPPs instead of a threonine. Indeed, Wang and Kantrowitz showed that a mutant version of the *E. coli* AP in which the catalytic serine was replaced by a threonine exhibited a 4000-fold decrease in k_cat_. [66]. Consistently, the catalytic site of human APs harbors three divalent cations: the two zinc ions previously described for ENPPs, maintaining identical organization and coordination, in addition to the Mg^2+^ ion. The existence of an extra divalent cation, coupled with the presence of the magnesium-binding helix, congests the enzyme’s catalytic site, thereby limiting its accessibility compared to ENPPs’ catalytic sites. The presence of this helix is also observed in the AP of *E. coli*, exhibiting identical folding and metal ion composition. Once again, a serine residue within the additional magnesium-binding helix facilitates magnesium coordination [65]. It is noteworthy that while an AP in *E. coli* shares 31.3% identity (59.8% similarity) over a 351 amino acid overlap with human TNAP, members of the ENPP family are notably absent. Overall, despite their structurally conserved catalytic center, members of the AP superfamily are able to bind divalent cations (Zn^2+^, Mg^2+^, Ca^2+^) that are specific to their family. Each family has its own set of key coordinating residues, granting each of them a unique divalent cation binding profile as well as a prominent sulfo- (ARSs) or phosphoester (APs and ENPPs) hydrolase activity.

## 5. Catalytic Binding Site and Substrate Accommodation

Despite a generally conserved catalytic center, significant reorganization of the substrate-binding sites is observed across the three families to optimally accommodate their respective substrates. This becomes evident when considering the enzyme surfaces in terms of charges and hydrophobicity. In the case of human arylsulfatases, six structures (ARSA, ARSB, IDS, ARSC, GALNS, and SGSH) provide insights into the organization of the catalytic center. However, no structures have been obtained with a natural substrate. Only two structures include a ligand: ARSA (PDB code: 1E2S, catalytic mutant C69A), where the structure was obtained by soaking in the presence of an artificial chromogenic substrate (p-nitrocatechol sulfate), and GALNS (PDB code: 4FDJ), where the structure was obtained by soaking in the presence of the catalytic product N-acetylgalactosamine [15,63]. However, the latter is observed in a non-productive orientation. Most of these enzymes exhibit a covalent intermediate with a covalently linked sulfate/phosphate to the catalytic residue [11,35,59]. As noted by Demydchuk et al. (2016), the catalytic site residues of these six enzymes are essentially conserved and nearly perfectly superimposable [16,26]. Generally, it is observed that the catalytic residues are accessible via a narrow pocket or cavity, located at the bottom of a crevice or a cleft (Figure 5E,F,G) [11,12,15,35,61]. Charges and hydrophobicity within this crevice dictate recognition specificity. For example, GALNS, which recognizes polyanionic substrates like the 6-sulfate groups of the N-acetyl-D-galactosamine 6-sulfate units of chondroitin sulfate and the D-galactose 6-sulfate units of keratan sulfate, and IDS, which recognizes as substrates the 2-sulfate groups of the L-iduronate 2-sulfate units of dermatan sulfate and heparan sulfate, both present a substrate-binding site that is highly positively charged (Figure 5E,F). ARSC, an enzyme that hydrolyzes sulfate groups from sulfated steroid precursors like dehydroepiandrosterone sulfate (DHEA-S) and estrone sulfate, has a binding site with one side that is positively charged and the other side that is hydrophobic (Figure 5G,H). To compensate for the absence of structures obtained in the presence of substrates, docking studies were also conducted. Bond et al. demonstrated, for instance, in their study of ARSB, an enzyme that cleaves the 4-sulfate groups of the N-acetyl-D-galactosamine 4-sulfate units found in chondroitin sulfate and dermatan sulfate, a binding site predominantly negatively charged on one side. Additionally, they observed the presence of some positive charges to secure the substrate’s end, along with a subtly polar groove designed to accommodate the remaining sugar chain [11].

In general, evolution has allowed aryl sulfatases to adapt to two major types of substrates by modifying the large groove surrounding the catalytic site, enabling these enzymes to recognize either extremely polar compounds, such as sulfated sugar polymers (e.g., GALNS, ARSB, SGSH, IDS, GNS, SULF1/2), or rather hydrophobic compounds like cerebroside sulfate (e.g., ARSA) or estrone sulfate (e.g., ARSC) [15,26,35,67,68]. Similar to aryl sulfatases, ENPPs exhibit an extremely conserved catalytic site, with an almost perfect conservation of residues involved in catalysis and cation binding. These residues are nearly perfectly superimposable (Figure 5D) [10]. Regarding substrate specificity, ENPPs can be categorized into three groups: those that can recognize polar compounds such as nucleotides or nucleotide derivatives with one, two, or three phosphate groups, including ENPPs 1, 3, 4, and 5; those that can recognize hydrophobic substrates with acyl chains and a single phosphate group, namely ENPPs 6 and 7; and finally, those with dual substrate specificity, represented by ENPP2 [10]. It is also noteworthy that ENPP6 can recognize short substrates like glycerophosphocholine [69].

As described by Borza et al., there is a significant conservation of the recognition site among ENPPs 1, 3, 4, and 5 [10]. These ENPPs have a deep polar groove, known as the nucleotide-binding slot, which accommodates the ribose and phosphate groups of their respective substrates (Figure 5I) [22,46,47,70].

They also have a constricted apolar zone, consisting of two aromatic residues (a tyrosine and a phenylalanine for ENPPs 1, 3, and 4, and two tyrosines for ENPP5). These residues facilitate π-π interactions with the nucleotide bases. The recognition site of ENPP2 is markedly different; it features a hydrophobic pocket for accommodating acyl chains and an open tunnel for allosteric effectors. Finally, ENPPs 5 and 6 have a simple groove capable of accommodating smaller substrates, with a reorganization of the base recognition zone, despite the sequence conservation and the presence of the two aromatic residues forming the constricted apolar zone [48,49]. Remarkably, these two aromatic residues are retained in ENPP6 but absent in ENPP7, even though the former does not initially appear to recognize nucleotides.

As mentioned earlier, APs have a catalytic site almost identical to that of ENPPs, except for the magnesium-binding helix. The catalytic residue and the residues interacting with zinc are almost perfectly superimposable, not only among AP members but also between APs and ENPPs (Figure 5H) [10]. In comparing the alkaline phosphatase of *E. coli* with an NPP from *Xanthomonas axonopodis*, Bobyr et al. reported that the catalytic sites of these two enzymes, particularly the coordination of Zn^2+^, were nearly identical, both in the absence of substrates and in the presence of vanadate, confirming similar mechanisms and structural organization [71]. However, the presence of the magnesium-binding helix and the resulting reorganization not only prevents the presence of the two aromatic residues necessary for base recognition but also leads to a complete closure of the substrate binding site, causing the APs to lose the nucleotide-binding slot (Figure 5I,J). This reorganization also results in a new mode of substrate recognition, where the substrate appears to present itself perpendicularly to the protein surface, as suggested by the presence of a PNP in the structure of PLAP (PDB code: 1ZED) (Figure 5J) [36]. In this conformation, the phosphate group is reoriented by approximately 90° compared to the phosphate groups of substrates present in the ENPPs. The authors further report that the structure of the enzyme complexed with 5′AMP after soaking exhibits a similar orientation. Despite the absence of density for the phosphate moiety and very low density for the ribose, the positioning of the base clearly confirms the same type of accommodation, with the substrate positioned perpendicular to the surface [36]. This arrangement could also explain the broader variety of substrates accepted by the APs, as the hydrolysis of the phosphate group does not require complete recognition of the backbone of the recognized molecules, which also explains why shorter substrates are preferentially accepted. Interestingly, when comparing the two existing structures of AP, two residues are found at the entrance of the catalytic site that cap zinc number 2. These residues are an aspartyl and a glutamyl in PLAP (D428 and E429) and an arginyl and a histidyl in TNAP (R450 and H451), suggesting that these “gatekeepers” may nonetheless contribute to some specificity in recognition. Notably, the residue E429 or H451 is replaced by a glycine residue (G429) in GCAP and a serine residue in IAP (S450) (Figure 5K). A study conducted by Watanabe et al. shows that PLAP and GCAP differ by only seven residues and that the simple replacement of the glycine residue at position 429 in GCAP with a glutamic acid residue results in sensitivity to inhibition by L-leucine comparable to that observed in PLAP. The second mutation with the strongest effects is the replacement of this same residue with a serine residue, increasing the enzyme’s resistance to inhibition by EDTA [72]. These results suggest that these “gatekeeper” residues likely play an important role in regulating the enzyme’s activity through substrate recognition, which explains the presence of three AP variants that are extremely similar in sequence yet essential in humans. Overall, these structural insights and predictive data indicate that substrate specificity observed within the AP superfamily can be explained by the differences in charge/hydrophobicity within the catalytic pocket. This will influence divalent cation binding, as well as favor interactions with the variable sidechains of sufo- and phosphoesters substrates. Moreover, the absence of a nucleotide-binding pocket in TNAP and other members of the AP family may explain their higher capacity for enzymatic promiscuity, as their activity is not limited to nucleotide dephosphorylation.

## 6. TNAP and ENPP, Diverging Enzymes with Complementary Biological Functions

As mentioned in the introduction, the two historically accepted TNAP substrates in vivo were PP_i_ and PLP. Hydrolysis of PP_i_ and PLP by TNAP indeed prevents the two main symptoms of TNAP deficiency in humans and mice: bone hypomineralization (PP_i_ is a potent mineralization inhibitor) and epileptic seizures (extracellular dephosphorylation of PLP is necessary for the cellular uptake of PL and for the PL-dependent synthesis of the inhibitory neurotransmitter GABA) [7]. Interestingly, one of these two substrates, PP_i_, is produced by ENPP1, indicating that at least one function, the control of pathophysiological mineralization, requires two enzymes of the AP superfamily. In fact, increasing evidence suggests that TNAP may exert other complementary functions with other ENPPs. In this last chapter, we will present the known functions of ENPP in humans and mice and how TNAP may participate in these functions. Actually, ENPP family members evolved in two main directions: towards the extracellular metabolism of nucleotides (ENPP1, ENPP3, ENPP4 and ENPP5), and towards the metabolism of choline-containing lipids (ENPP2, ENPP6 and ENPP7) (Table 2).

Among all ENPP members, *ENPP1* is probably the gene whose variants are associated with the broader spectrum of clinical symptoms [73]. ENPP1 mutations are associated with different types of ectopic calcifications, resulting mainly, but maybe not exclusively, from reduced PP_i_ production. These ectopic calcifications range from vascular calcifications in generalized arterial calcification of infancy (GACI) [74] and *pseudoxanthoma elasticum* [75] to skin calcifications in Cole disease [76], and ossifications in tendons and ligaments in diffuse idiopathic skeletal hyperostosis (DISH) and ossification of the posterior longitudinal ligament (OPLL). *ENPP1* mutations are also associated with autosomal recessive hypophosphatemic rickets type 2 ARHR2 [77,78], due to high levels of the phosphaturic hormone FGF23 of obscure origin. In addition, variants in the somatomedin (SMD) domains can also induce metabolic disorders. A specific polymorphism in the ENPP1 SMD domain (K121Q) is strongly associated with early-onset obesity, insulin resistance and cardiovascular mortality risk [79]. This association seems to result from the inhibition of the catalytic activity of the insulin receptor by ENPP1 interaction [80]. Finally, ENPP1 also hydrolyzes cyclic GMP-AMP (cGAMP), a molecule synthesized by cyclic GMP-AMP synthase from ATP and GTP after the recognition of cytoplasmic DNA, to activate the immune response by binding to stimulator of interferon gene (STING) on endoplasmic reticulum membrane [81]. ENPP3 appears predominantly expressed in mast cells and basophils. ENPP3-deficient mice are more susceptible to allergic responses, due to reduced autocrine/paracrine ATP hydrolysis by mast cells and basophils [82]. In addition, and like ENPP1, ENPP3 was recently identified as a hydrolase of cGAMP, acting at the cell membrane in a paracrine fashion to modulate STING-associated immune responses [83]. ENPP3 expression pattern is distinct from that of ENPP1, and ENPP3 is more active at acidic pH than ENPP1, which might confer an advantage in an acidic tumor microenvironment. Finally, ENPP3 was proposed to participate in the Golgi apparatus for protein glycosylation, by hydrolyzing UDP-GlcNAc into AMP and GlcNAc-1-P [84]. ENPP4 has been proposed to hydrolyze the dinucleotides diadenosine Ap3A and Ap4A at the endothelium surface [85]. These dinucleotides are released in the blood upon platelet degranulation. Their hydrolysis by ENPP4 would locally generate ADP, which would help sustain degranulation by binding to purinergic P2Y receptors P2Y1 and P2Y1 at the platelet surface. ENPP5 appears to hydrolyze nicotinamide adenine dinucleotide (NAD) into AMP and nicotinamide mononucleotide (NMN) [47]. The consequences of this function in mice or humans are unknown.

Besides these ENPPs playing a role in nucleotide metabolism, ENPP2, ENPP6 and ENPP7 evolved to participate in the extracellular hydrolysis of choline-associated lipids into choline. ENPP2 (aka autotaxin) is the only secreted ENPP member, but it can bind to the membrane of target cells through the binding of SMD domains with integrins [86]. The main substrate of ENPP2 is lysophosphatidylcholine (LPC), which is cleaved by ENPP2 as a lyso-PLD manner to release choline and lysophosphatidic acid (LPA). Although choline is an important metabolic precursor for different pathways, the main function of ENPP2 may rely on the action of LPA on G protein coupled receptors, to trigger a wide variety of molecular signals [86]. ENPP2-deficient mice show embryonic lethality, while heterozygous deficient mice appear healthy despite having half LPA plasma levels. In humans, ENPP2 is thought to be primarily important in cancer. Like ENPP2, ENPP6 can hydrolyze LPC, but like a phospholipase C, releasing phosphocholine and not choline [49]. ENPP6 is also active on glycerophosphocholine [49]. ENPP6 is expressed in the brain and in liver endothelial cells. ENPP6 appears to participate in extracellular choline production to allow oligodendrocytes in the brain and hepatocytes in the liver to uptake choline and produce phosphatidylcholine [49]. ENPP7 (aka alkaline sphingomyelinase) is predominantly expressed in the intestinal tract to hydrolyze sphingomyelin to phosphocholine and ceramide during digestion [87]. Deficiency in ENPP7 is associated with defective sphingomyelin digestion.

Strikingly, although TNAP is, unlike ENPP, a phosphatase, it seems to participate in the same metabolic pathways as ENPPs. Concerning nucleotide metabolism, we already mentioned that the production of PP_i_ from ATP by ENPP1 is followed by the hydrolysis of PP_i_ by TNAP. Moreover, like ENPP1 and ENPP3, TNAP may participate in ATP hydrolysis in some particular cells and situations. For instance, dephosphorylation of ATP by TNAP in neurons was proposed to allow the growth of axons [88]. In addition, TNAP hydrolysis of AMP into adenosine may help resolve ATP-associated inflammation in neutrophils [5,89]. Impairment of these nucleotidase functions in patients with HPP may induce epileptic seizures [90] and osteomyelitis [91]. Whether, besides ATP and AMP, TNAP dephosphorylates other nucleotides produced by ENPP1/2/4/5 is not known but merits investigation.

With regard to extracellular choline metabolism, TNAP likely participates with ENPP2/6/7 in choline release from choline-associated lipids. Indeed, TNAP is likely the phosphatase that dephosphorylates extracellular phosphocholine released by ENPP6 and ENPP7 from glycerophosphocholine and sphingomyelin, respectively [49,87]. In the brain, ENPP6 and TNAP are probably the enzymes that, respectively, hydrolyze glycerophosphocholine into phosphocholine [49] and phosphocholine into choline [92]. ENPP6- and TNAP-deficient mice show myelination problems [49,93], which may result from insufficient amounts of choline to produce sphingomyelin. The same complementary function of ENPP6 and TNAP also likely takes place in the liver. Phosphocholine dephosphorylation by TNAP in the liver may allow cellular choline uptake [94,95]. Like wildtype mice fed with a choline-deficient diet, ENPP6 and TNAP-deficient mice develop liver steatosis, which likely results from the impairment of phosphatidylcholine synthesis in hepatocytes and insufficient production of very low-density lipoproteins [49,96]. Whether a similar complementary function exists for ENPP7 and TNAP is unknown but possible. Interestingly, ENPP7 is predominantly expressed in the gut, where it hydrolyzes sphingomyelin into phosphocholine during digestion. The AP in the intestinal epithelium is not TNAP but IAP, which was reported to hydrolyze phosphocholine more potently than TNAP [97]. Overall, TNAP and ENPPs, by their complementary biological function, participate in the processing of numerous extracellular mono-, di- and triphosphate nucleotides, lipids and other similar metabolites. Their activity is crucial for cell homeostasis, as it ensures the internalization/processing of precursors involved in calcification, cell signaling, glycerophospho- and phosphosphingolipid synthesis, immune response and inflammation.

## 7. Conclusions

The alkaline phosphatase superfamily in humans comprises two major categories of activity, characterized by a conserved central fold and a catalytic site organization with many similarities. Arylsulfatases form a relatively conserved first group, while ENPPs and APs make up a relatively homogeneous second group. Despite the high conservation of catalytic residues and those involved in interaction with divalent cations at the catalytic site, each group has evolved to recognize a wide variety of substrates. This evolution is achieved through the fine adaptation and reorganization of substrate recognition sites, illustrated by changes in charge, hydrophobicity, and accessibility to the catalytic residues. One of the most interesting observations is the structural evolution of ENPPs and APs, which has led to a continuity in substrate recognition and functional convergence. Specifically, the activity of ENPPs produces reaction products such as PP_i_ and/or monophosphate nucleotides, which are then processed by APs. Additionally, some ENPPs share specificities with APs. For example, ENPP6, like APs, has a GPI anchor at the C-terminus and the ability to form AP-like dimers. From this perspective, APs are the most recent additions to this large family, distinguished by their unique feature, a magnesium ion, and its associated binding helix.

## Figures and Tables

**Table 1:** Members of the AP, ENPP and ARS subfamilies in humans and mice. Some differences exist between the two species. For instance, while there is a single intestinal alkaline phosphatase (IAP) in humans, two IAPs are present in mice, encoded by two different genes.

Human Protein	Human Gene	Mouse Protein	Mouse Gene
Tissue-nonspecific alkaline phosphatase (TNAP)	*ALPL*	TNAP	*Alpl (Akp2)*
Intestinal alkaline phosphatase (IAP)	*ALPI*	Global intestinal alkaline phosphatase (gIAP)	*Akp6*
Placental alcaline phosphatase (PLAP)	*ALPP*	Duodenal-specific intestinal alkaline phosphatase (dIAP)	*Akp3*
Germ cell alkaline phosphatase (GCAP)	*ALPG*	Embryonic alkaline phosphatase (EAP)	*Akp5*
Ectonucleotide pyrophosphatase/phosphodiesterase 1 (ENPP1)	*ENPP1*	ENPP1	*Enpp1*
Ectonucleotide pyrophosphatase/phosphodiesterase 2 (ENPP2)	*ENPP2*	ENPP2	*Enpp2*
Ectonucleotide pyrophosphatase/phosphodiesterase 3 (ENPP3)	*ENPP3*	ENPP3	*Enpp3*
Ectonucleotide pyrophosphatase/phosphodiesterase 4 (ENPP4)	*ENPP4*	ENPP4	*Enpp4*
Ectonucleotide pyrophosphatase/phosphodiesterase 5 (ENPP5)	*ENPP5*	ENPP5	*Enpp5*
Ectonucleotide pyrophosphatase/phosphodiesterase 6 (ENPP6)	*ENPP6*	ENPP6	*Enpp6*
Ectonucleotide pyrophosphatase/phosphodiesterase 7 (ENPP7)	*ENPP7*	ENPP7	*Enpp7*
Arylsulfatase A	*ARSA*	Arylsulfatase A	*Arsa*
Arylsulfatase B	*ARSB*	Arylsulfatase B	*Arsb*
Arylsulfatase C	*ARSC*	Arylsulfatase C	*Arsc*
Arylsulfatase D	*ARSD*		
Arylsulfatase E	*ARSE*	Arylsulfatase E	*Arse*
Arylsulfatase F	*ARSF*		
Arylsulfatase G	*ARSG*	Arylsulfatase G	*Arsg*
Arylsulfatase H	*ARSH*		
Arylsulfatase I	*ARSI*	Arylsulfatase I	*Arsi*
Arylsulfatase J	*ARSJ*	Arylsulfatase J	*Arsj*
Arylsulfatase K	*ARSK*	Arylsulfatase K	*Arsk*
Galactosamine 6-sulfatase	*GALNS*	Galactosamine 6-sulfatase	*Galns*
Glucosamine 6-sulfatase	*GNS*	Glucosamine 6-sulfatase	*Gns*
Sulphoglucosamine sulphohydrolase	*SGSH*	Heparan N-sulfatase	*Sgsh*
Iduronate 2-sulfatase	*IDS*	Iduronate 2-sulfatase	*Ids*
Sulfatase 1	*SULF1*	Sulfatase 1	*Sulf1*
Sulfatase 2	*SULF2*	Sulfatase 2	*Sulf2*

**Table 2:** AP superfamily members, their known or suspected substrates and products, molecular functions, the phenotype of deficient mice, and the impact of gene mutations/polymorphisms in humans. ENPPs are presented according to their metabolic involvement (ENPP1/3/4/5 in nucleotide metabolism, and ENPP2/6/7 in choline-associated lipid metabolism). ARHR2: autosomal recessive hypophosphatemic rickets type 2; DISH: diffuse idiopathic skeletal hyperostosis; GABA: gamma-aminobutyric acid; LPA: lysophosphatidic acid; OPLL: ossification of the posterior longitudinal ligament; P_i_: inorganic phosphate; PL: pyridoxal; PLP: pyridoxal phosphate; PP_i_: inorganic pyrophosphate; STING: stimulator of interferon gene.

	Known/Suspected Substrates	Products	Molecular Consequences	Phenotype of Deficient Mice	Impact of Gene Mutations/Polymorphisms in Humans
**ENPP1**	ATP	AMP and PP_i_	Reduced levels of PP_i_, a calcification inhibitor	Ectopic calcifications in arteries, tendons and ligaments	Generalized arterial calcification of infancy (GACI) and pseudoxanthoma elasticum, charcterized by ectopic calcifications
					Ossification of the posterior longitudinal ligament (OPLL) or diffuse indiopathic skeletal hyperostosis (DISH)
					Cole disease, with sometimes calcification of the skin and tendons
	Obscure	Obscure	Elevated FGF23 levels and Pi excretion of obscure origin	Hypophosphatemia	Autosomal recessive hypophosphatemic rickets type 2 (ARHR2)
	Variants in the SMD domains, in particular K121Q, leading to reduced insulin receptor activity resulting from increased interaction between the SMB2 domain of ENPP1 and insulin receptor			Protection against obesity and diabetes	Childhood obesity and type 2 diabetes
	cGAMP	GMP and AMP	Decreased STING activation, immune checkpoint	Increased inflammation	Poorly known
**ENPP3**	ATP	ADP	Decreased inflammation in mast cells and basophils	Chronic allergic pathologies	Poorly known
	cGAMP	GMP and AMP	Decreased STING activation, immune checkpoint	Increased inflammation	
	UDP-GlcNAc	GlcNAc-1-P and AMP	Inhibition of protein glycosylation in Golgi apparatus	Not described	
**ENPP4**	Ap3A	ADP and AMP	Stimulation of platelet degranulation	Not described	Poorly known
	Ap4A	ATP and AMP			
**ENPP5**	NAD+	AMP and NMN	Not known	Not known	Not known
**ENPP2**	Lysophosphatidylcholine	LPA	Signals through at least 6 G protein coupled receptors	Lethal during embryon life	Poorly known
		Choline	Multiple metabolic functions		
**ENPP6**	Glycerophosphocholine	Phosphocholine	Choline precursor	liver steatosis and hypomyelination	Poorly known
**ENPP7**	Sphingomyelin	Phosphocholine	Choline precursor	Altered sphingomyelin digestion	Poorly known
**TNAP**	PP_i_	P_i_	High bone levels of PP_i_, an inhibitor of mineralization	Bone hypomineralization	More than 400 known mutations in ALPL gene leading to hypophosphatasia, mainly characterized by bone hypomineralization, auto-inflammations, neurodevelopmental defects, and epileptic seizures
	PLP	PL	Reduced PL uptake and GABA production in the brain	Epileptic seizures	
	ATP and/or AMP	Adenosine	Neuronal transmission; inflammation	ATP-induced seizures	
	Phosphocholine	Choline	Reduced phosphatidylcholine production	Liver steatosis; myelination defects	

## Data Availability

No new data were created or analyzed in this study. Data sharing is not applicable to this article.
